# The assessment of angiogenesis and fibroblastic stromagenesis in hyperplastic and pre-invasive breast lesions

**DOI:** 10.1186/1471-2407-8-88

**Published:** 2008-04-02

**Authors:** Kitty Pavlakis, Irene Messini, Thomas Vrekoussis, Petros Yiannou, Dimitrios Keramopoullos, Niki Louvrou, Theodoros Liakakos, Efstathios N Stathopoulos

**Affiliations:** 1 Pathology Department, Medical School, University of Athens, Athens, Greece; 2 Pathology Department "IASO" Hospital, Athens, Greece; 3 Pathology Department, Medical School, University of Crete, Heraklion, Crete, Greece; 4 Breast Surgery Clinic "IASO" Hospital, Athens, Greece; 5 3rd Surgical Clinic, University Hospital "ATTIKON", Haidari, Athens, Greece

## Abstract

**Background:**

To investigate the changes of the neoplastic microenvironment during the different morphological alterations of hyperplastic and pre-invasive breast lesions.

**Methods:**

78 in situ ductal carcinomas of all degrees of differentiation, 22 atypical ductal hyperplasias, 25 in situ lobular carcinomas, 18 atypical lobular hyperplasias, 32 ductal epithelial hyperplasias of usual type and 8 flat atypias were immunohistochemically investigated for the expression of vascular endothelial growth factor (VEGF), smooth muscle actin (SMA) and CD34, while microvessel density (MVD) was counted using the anti-CD31 antibody.

**Results:**

VEGF expression was strongly correlated with MVD in all hyperplastic and pre-invasive breast lesions (p < 0.05). Stromagenesis, as characterized by an increase in SMA and a decrease in CD34 positive myofibroblasts was observed mostly around ducts harboring high grade in situ carcinoma and to a lesser extent around moderately differentiated DCIS. In these two groups of in situ carcinomas, a positive correlation between MVD and SMA (p < 0.05) was observed. On the contrary, CD34 was found to be inversely related to MVD (p < 0.05). No statistically significant changes of the stromal fibroblasts were observed in low grade DCIS neither in any of the other lesions under investigation as compared to normal mammary intra- and interlobular stroma.

**Conclusion:**

Angiogenesis is observed before any significant fibroblastic stromagenesis in pre-invasive breast lesions. A composite phenotype characterized by VEGF positive epithelial cells and SMA positive/CD34 negative stromal cells, is identified mostly in intermediate and high grade DCIS. These findings might imply for new therapeutic strategies using both anti-angiogenic factors and factors selectively targeting tumor stroma in order to prevent the progression of DCIS to invasive carcinoma.

## Background

There is considerable body of evidence from many investigations, that the stroma immediately adjacent to a tumor is not a passive structural element that elicits an immune response in an attempt to reject the tumor, but an element that actively participates and contributes to tumor progression [1-5]. Furthermore, it has been proposed that carcinogenesis does not result from epithelial or stromal mutations alone, but rather from the loss or breakdown of biological structures induced by perturbed stromal-epithelial interactions [6,7]. Recent data have demonstrated that the tumor microenvironment facilitates metastatic spread by eliciting reversible changes in the phenotype of cancer cells [8]. A key cell type involved in the development of the tumor-promoting reactive stroma appears to be the myofibroblast [9,10].

It has been shown that carcinoma cells have the capacity to induce normal fibroblasts to turn into the reactive myofibroblastic phenotype. Substances which are synthesized by these myofibroblasts such as collagen I and II, fibronectin isoforms, tenascin and versican as well as proteases that are expressed by myofibroblasts such as metalloproteinases (MMPs), urokinase plasminogen activator and fibroblast activating factor (FAP) induce a remodeling of the extracellular matrix (ECM) that could stimulate cancer growth and migration. Moreover, myofibroblasts secrete growth factors, such as the connecting tissue growth factor (CTGF) and the transforming growth factor beta-1 (TGF beta1) which have potent angiogenic activities [11].

The mesenchymal fibroblastic stroma reactions that are induced by, and regulated parallel to epithelial neoplastic transformation, are termed "stromagenesis".

In the mammary gland, based on experimental studies, Cuhiermann [12] defined three stages of stromagenesis: "normal", "primed" and "activated". The term *normal stroma *stands for a neoplastic progression-restraining environment, the term *primed stroma *for a permissive and supportive landscape for tumor progression and the term *activated stroma *for the advanced neoplastic microenvironment. During breast stromagenesis there is downregulation of some stromal genes, such as CD34 and upregulation of other genes indicative of myofibroblastic differentiation, such as smooth muscle actin (SMA) [13].

On the other hand, another key step in tumor progression is the formation of new blood vessels from a pre-existing vascular network, known as angiogenesis [14,15]. It has been demonstrated that an important event in the process of angiogenesis is the recruitment of endothelial progenitor cells to sites of the new vessel formation with subsequent differentiation into mature endothelial cells. This phenomenon is induced by angiogenic chemokines produced by the neoplastic cells [16]. In the mammary gland, the formation of a vascular stroma was found to precede invasion [17,18], while higher levels of angiogenic marker molecules seem to be associated with poor prognosis [19]. It has been postulated that tumor cells do not invade into normal breast stoma but rather into a richly vascular stroma that they have induced. This process of neovascularization is driven by growth factors released into the stroma by tumor cells and immune cells. One of these growth factors, the vascular endothelial cell growth factor (VEGF) was found to be overexpressed in neoplastic intraductal and intralobular breast lesions as compared to normal glandular structures [20]. Yet, the results of the relationship between VEGF expression and the degree of vascularization in pre-invasive breast lesions are controversial [20,21]. In the present study we sought to determine by immunohistochemistry, the steps of periglandular stromal transformation in all types of pre-invasive breast lesions in respect of their angiogenic and stromagenic potential.

## Methods

This study was approved by the Ethical Committee of "IASO" Hospital, Athens, Greece.

### Specimens

Archival material from the files of the Department of Pathology, "IASO" Hospital was used for the study. Four 4μ thick sections were cut from the paraffin blocks of 78 in situ ductal carcinomas of all degrees of differentiation (21 low grade/DCIS-L, 24 intermediate grade/DCIS-I and 33 high grade/DCIS-H) classified according to Holland et.al [22], 9 atypical ductal hyperplasias/ADH, 25 in situ lobular carcinomas/LCIS, 11 atypical lobular hyperplasias/ALH, 32 ductal epithelial hyperplasias of usual type/HUT and 8 flat atypias/FA. Consecutive sections were used in order to obtain a better comparison between morphology and protein expression. Normal mammary parenchyma obtained from 10 women who underwent breast reduction was also analyzed.

### Immunohistochemistry

Immunohistochemical staining was performed using the EnVision+ System-HRP. Tissue sections were deparaffinized, rehydrated and treated with a hydrogen peroxide solution for 10 min to quench endogenous peroxidase. Sections were then heated in a microwave oven at 600 W for 30 min in Target Retrieval Buffer, pH = 6.0 (DakoCytomation). After cooling for 20 min, they were incubated with the primary antibody (rabbit anti-human VEGF, dilution 1:100, Oncogene Research Products, mouse anti-human CD31, clone JC70A, dilution 1:80, Dako Corporation, mouse anti-human CD34, dilution 1:50, clone QBEnd/10, Neomarkers and mouse anti-human a-SMA, dilution 1; 600, Dako) for 1 hour at room temperature and then incubated for 45 min with the anti-mouse HRP labeled polymer, included in the EnVision Kit. Finally sections were treated with a diaminobenzidine (DAB) chromogenic substrate for 10 min, counterstained with Mayer's hematoxylin, dehydrated and coverslipped.

### Evaluation of microvascular density (MVD)

Images were captured using a Zeiss Axiolab microscope (Carl Zeiss Jena GmbH, Jena, Germany) with a mechanical stage, fitted with a Sony-iris CCD videocamera (Sony Corporation, Tokyo, Japan). The video camera was connected to a Pentium II personal computer loaded with the Image Scan Software (Jandel Scientific, Erkrath, Germany). In each case, 3–5 optical fields × 200 were selected from the periphery of each gland harboring an intraepithelial proliferation. Counts were done at a rim of 500 μm in width from the periphery of each structure. In normal breast, each duct-lobular structure was considered as one separate entity and the whole inter- and intralobular stroma was evaluated. Pictures were stored as JPEG files [(1550 × 1070 pixels, 16.7 million colors (24-bit)]. Single endothelial cells or clusters of endothelial cells, with/without obvious lumen, positive for CD31 were considered as individual vessels. In each vessel, the outline was interactively identified. The presence of blood cells or fibrin without any detectable endothelial cells was not considered sufficient to define a microvessel. Areas with a dense leukocytic or hemorrhagic infiltration were excluded. Vessels with muscular wall were not counted; however, there was no restriction regarding the size of the countable microvessels, so as not to underestimate longitudinal sections or bifurcations of microvessels.

### Evaluation of labeling

Positive staining of VEGF was detected in the cytoplasm of the epithelial cells. Immunoreactivity for VEGF was evaluated semiquantitatively by two independent pathologists (K.P. and P.Y.) with reference to both the staining intensity and the positively stained area. Staining intensity was scored as follows: 0, none; 1, weak; 2, moderate; 3, strong. The positively stained area was expressed as the percentage of the whole area under evaluation and scored as: 0, none; 1: 1–25%, 2: 26–50%, 3: 51–75% and 4: 76–100%). The product was then graded as 0–2 = low, 3–5 = intermediate and 6–7 = high.

Fibroblastic reactivity for CD34 and a-SMA was evaluated using the same protocol as for angiogenesis. It was recoded as positive (+), negative (-) or intermediate (+/-) where the staining was focal.

### Statistical analysis

The mean microvascular density was calculated for each histological group. For each study group, the positivity index for each staining was calculated as the ratio of specimens being strongly or intermediately stained over the sample population. The positivity index of each histological group was then correlated to the group's mean MVD, using the Pearson r test. Both SMA and CD34 expression pattern for every histological entity in this study were compared with the relevant expression of the normal breast tissue. Further analysis was performed on the DCIS samples as these seem to differ from normal tissue. For the comparisons between normal breast tissue samples with the other histology groups, the Fisher exact probability test for contingency tables larger than 2 × 2, was applied. Every observation presented with p < 0.05 was considered as significant.

## Results

In the normal breast, all duct-lobular units exhibited CD34 positive fibroblasts while there was no expression of SMA myofibroblasts with the exception of four cases showing weak focal staining. Nearly the same pattern of fibroblastic expression was observed around glands showing HUT, ADH, FA, ALH, LCIS and DCIS-L, as shown in Table 1. A dramatic decrease of CD34 expression of fibroblasts and acquisition of SMA was mostly observed around ducts harboring DCIS-I and DCIS-H. Interestingly, in four cases of DCIS-I and three cases of DCIS-H a homogenous pattern of staining was observed with fibroblasts being strongly positive to both CD34 and SMA.

**Table 1:** Presentation of the positivity indices (PI) of CD34, SMA and VEGF among the histological groups of study, in relation to MVD. CD34 and SMA reactivities are graded as negative (n), intermediate (i) and positive (p). VEGF expression is evaluated by the product of staining intensity score multiplied with the percentage of the staining area score. This product is graded as low (L), intermediate (I) and high (H).

**STAINING**	**CD34 (n)**	**CD34 (i)**	**CD34 (p)**	**CD34 (PI)**	**SMA (n)**	**SMA (i)**	**SMA (p)**	**SMA (PI)**	**VEGF (L)**	**VEGF (I)**	**VEGF (H)**	**VEGF (PI)**	**MVD **Mean(vessel number/mm^2^) ± SD
NORMAL *(n = 20)*	0	0	20	1	16	4	0	0.2	18	2	0	0.1	86 ± 3
HUT *(n = 32)*	0	0	32	1	30	2	0	0.062	32	0	0	0	79 ± 7
ADH *(n = 22)*	1	1	20	0.954	21	1	0	0.045	13	3	6	0.409	119 ± 10
FA *(n = 8)*	0	1	7	1	7	0	1	0.125	4	2	2	0.5	114 ± 9
DCIS-L *(n = 21)*	0	4	17	1	14	6	1	0.333	5	12	4	0.761	121 ± 13
DCIS-I *(n = 24)*	8	5	11	0.667	0	11	13	1	5	14	5	0.791	140 ± 32
DCIS-H *(n = 33)*	25	5	3	0.242	0	0	33	1	6	11	16	0.818	146 ± 38
ALH *(n = 11)*	0	0	11	1	11	0	0	0	11	0	0	0	109 ± 5
LCIS *(n = 25)*	0	1	24	1	22	3	0	0.12	15	9	1	0.4	111 ± 7

Taking into consideration the fact that, in DCIS-I and DCIS-H, the SMA positivity index was higher, while the CD34 positivity index was lower than the rest groups of study (Table 2), it was necessary to define whether this difference was of significance. As shown (Table 3) there was no statistical difference between the normal samples and the histological groups HUT, ADH, FA, ALH, LCIS and low grade DCIS (DCIS-L) regarding SMA and CD34 staining. On the contrary both SMA and CD34 expression patterns were revealed significantly different on DCIS-I and DCIS-H when compared to normal breast tissue samples. Additionally, the analysis performed on the DCIS samples alone, showed that both SMA and CD34 staining patterns were significantly different between the three groups of study DCIS-L, DCIS-I and DCIS-H (Table 3).

**Table 2:** Comparison between normal breast tissue and the histological groups of study according to SMA and CD34 staining. It is shown that only DCIS-I and DCIS-H differ significantly from normal breast tissue. (The significant p values are underlined)

	**HUT**	**ADH**	**FA**	**ALH**	**LCIS**	**DCIS-L**	**DCIS-I**	**DCIS-H**
***Normal (SMA)***	P = 0.18	P = 0.17	P = 0.21	P = 0.26	P = 0.68	P = 0.59	P < 10^-9^	P < 10^-14^
***Normal (CD34)***	P~1.0	P~1.0	P = 0.28	P~1.0	P~1.0	P = 0.11	P < 10^-4^	P = 0.002

**Table 3:** Comparison between the DCIS groups according to SMA and CD34 staining. It is shown that the three groups differ significantly from each other both in SMA and CD34 staining.

	**DCIS-H**	**DCIS-I**
***DCIS-L (SMA)***	P < 10^-13^	P < 10^-6^
***DCIS-I (SMA)***	P < 10^-4^	
***DCIS-L (CD34)***	P < 10^-10^	P < 10^-2^
***DCIS-I (CD34)***	P < 10^-3^	

Serial sections were taken from each case and were stained with VEGF and CD31 for the assessment of angiogenesis. It has been shown that the highest VEGF levels were observed in DCIS-H, while high and intermediate VEGF values were encountered in DCIS-I and DCIS-L and to a lesser extent in FA. All other morphological entities under investigation presented mostly low VEGF expression. The evaluation of angiogenesis with anti-CD31 antibody revealed that high microvessel counts were strongly associated with DCIS-I and DCIS-H, with a mean of 140 ± 32 and 146 ± 38 respectively and to a lesser extent with DCIS-L, ADH, FA, LCIS and ALH (mean values 121 ± 13, 119 ± 10, 114 ± 9, 111 ± 7 and 109 ± 5 respectively). Microvessel counts around normal breast ducts and lobules and around ducts harboring HUT were found to be significantly lower (Table 1).

**Figure 1:**
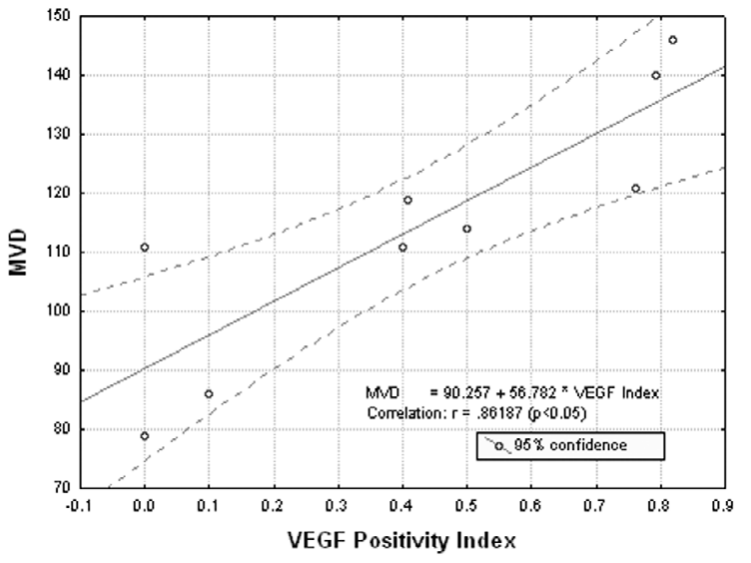
**Correlation analysis between VEGF positivity index and microvascular density (MVD).** A significantly positive correlation between VEGF staining and microvascular density, is showed (p < 0.05).

**Figure 2:**
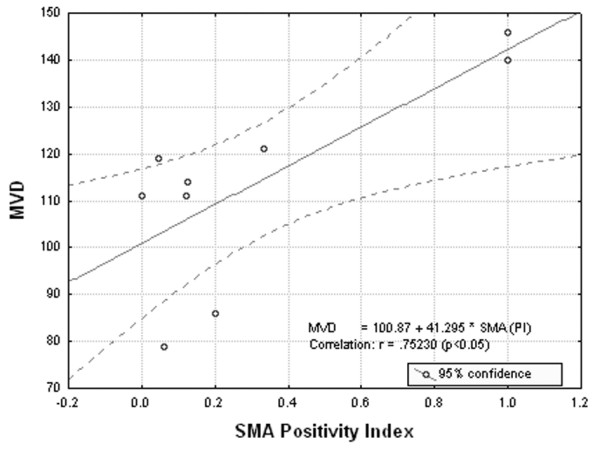
**Correlation analysis between SMA positivity index and microvascular density (MVD). **A significantly positive correlation between SMA staining and microvascular density, is showed (p < 0.05).

**Figure 3:**
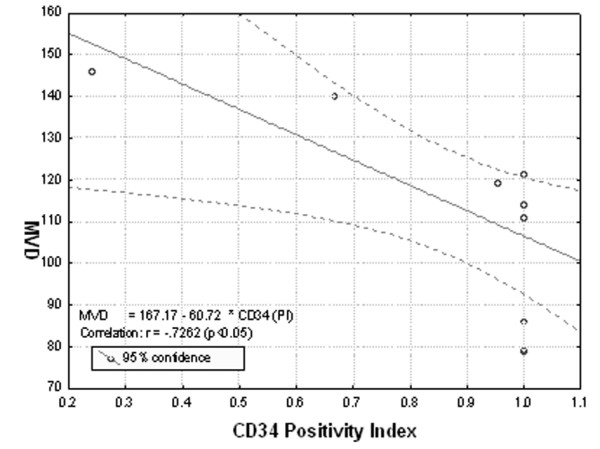
**Correlation analysis between CD34 positivity index and microvascular density (MVD).** A significantly negative correlation between CD34 staining and microvascular density, is showed (p < 0.05).

There was a strong correlation between VEGF, SMA, CD34 and microvascular density. As shown in Fig. 1, VEGF expression was strongly correlated with MVD (p < 0.05). A positive correlation was revealed between SMA staining and MVD (p < 0.05) (Fig. 2). CD34 staining was proved to be inversely related to MVD (p < 0.05) (Fig. 3).

## Discussion

Over the past decade the "tissue-microenvironment" concept of malignancy has gained space over the epitheliocentric view of tumorigenesis [23,24]. The emerging concept from several investigations implies that loss of tissue architecture or derangement of cell adhesion would drive malignant behavior of cells within a tissue, even in the absence of primary genetic mutations. This phenomenon could be induced by perturbed stromal-epithelial interactions. Moreover, a repression of the malignant phenotype of genetically aberrant cells has been observed after restoration of tissue organization. The Tissue Organization Field Theory proposes that proliferation is "the default state of all cells" [25,26].

In breast carcinogenesis, several experimental studies have focused on tumor-microenvironment interactions. Using laser capture microdiscection, Kurose et. al. [2] identified frequent loss of heterozygocity (LOH) in both neoplastic epithelial and stromal compartment. The most important, they noted that genetic alterations occurred in the epithelial compartment on the earlier steps, followed by LOH in the stromal compartments, which indicates that the genetic alterations in the epithelia precede the ones in the stroma. Another group of investigators also supports the concept of stromal-epithelial interactions in the development and progression of mammary neoplasia but in their study the genetic alterations of the stromal cells were found to precede genotypic changes in the epithelial cells [27]. It seems that the switch from fibroblasts to myofibroblasts simulates cancer progression via an epithelial-mesenchymal transition [28].

**Figure 4:**
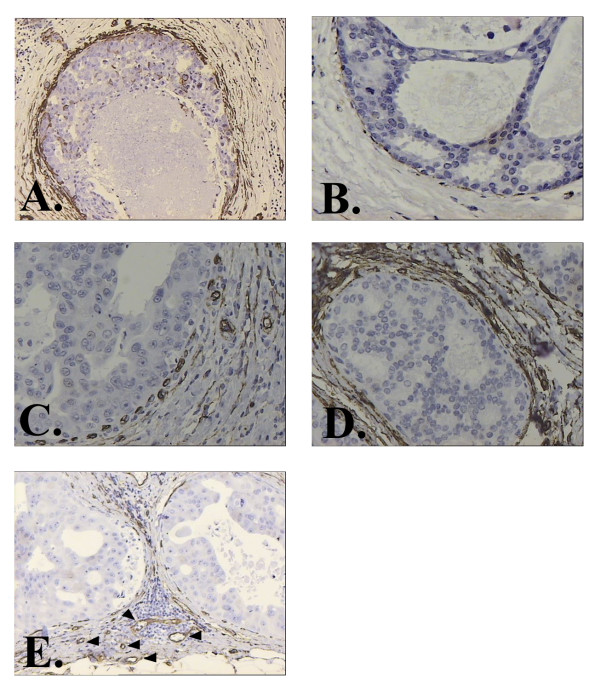
**Study of SMA expression among high and low grade ductal carcinoma in situ, revealed that there are no SMA positive myofibroblasts around DCIS-L (B) while around high grade DCIS, SMA positive myofibroblasts are identified (A).** The opposite phenomenon is observed regarding CD34 expression upon stroma fibroblasts. It is shown that in high grade DCIS there is a dramatic decrease of CD34 positive fibroblasts (C) as opposed to the CD34 positive stroma fibroblasts of DCIS-L (D). CD 31 staining showing a high microvessel density of the stroma adjacent to a DCIS-H (E). Arrowheads showing CD 31 positively stained vessels. Magnification × 100.

The results of our study are more consistent with those of the former investigators. Stromal changes as identified immunohistochemically by loss or dramatic increase of CD34 positive fibroblasts and detection of SMA-reactive myofibroblasts were observed mostly around ducts harboring moderately or poorly differentiated in situ carcinoma (Fig. 4A–D). This finding might imply that stromal changes are a late and possibly surrogate event in a process of multistage carcinogenesis and local invasion. Yet, recent studies on the molecular evolution of breast cancer have raised the possibility that moderately and poorly differentiated ductal carcinomas in situ might represent clonal proliferations of different cytogenetic clones [29,30]. The steps from atypical epithelial hyperplasia, to intraductal and then to invasive carcinoma are not any more considered as being always part of a linear progression [30]. If this is true, one could speculate that tumor stroma, in order to be induced, needs to interact with epithelial neoplastic cells harboring distinct genetic alterations.

Low grade ductal carcinoma in situ and in situ lobular carcinoma are now believed to belong to the same low-grade pathway of progression to invasive carcinoma [31]. The cytogenetic similarities encountered in these two morphologically distinct entities, namely losses in 16q and gains in 1q chromosomes, might justify for the absence in our study, of tumor-microenvironment alterations, for both lesions, regarding the expression of CD34 and SMA. Moreover, no statistical difference was found between the normal samples and the histological groups HUT, ADH, FA and ALH regarding SMA and CD34 staining pattern.

**Figure 5:**
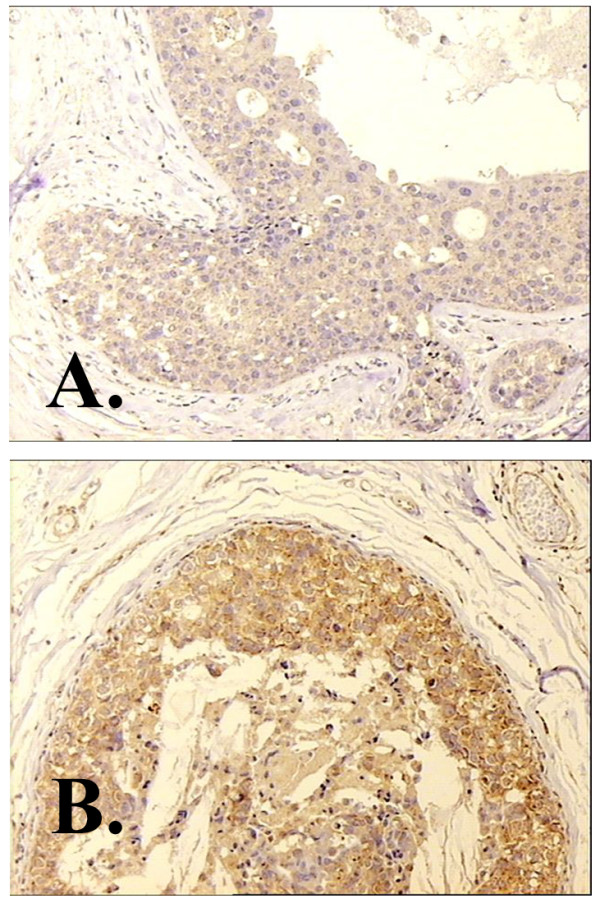
**(A) Intermediate cytoplasmic VEGF expression in a DCIS-L (intensity 1, percentage 4, score 5) and (B) Strong cytoplasmic VEGF expression in a DCIS-H (intensity 3, percentage 4, score 7).** Magnification ×100.

In our study, angiogenesis, as evaluated by the measurement of MVD, was observed before any significant fibroblastic stromagenesis, since a high microvessel count was found in all in situ ductal carcinomas and to a lesser extent around glands harboring ADH, FA, LCIS and ALH, while stromagenesis was mostly observed around ducts harboring DCIS-I and DCIS-H. Moreover, the pattern of neovascularization was characterized by a diffuse increase in stromal vascularity between ducts (Fig. 4E). It has been suggested that the angiogenic pathway corresponding to the diffuse pattern is more likely to be controlled by VEGF [32]. Indeed, in our study, there was a statistically significant relationship between VEGF immunohistochemical expression and the degree of MVD. Pre-invasive lobular lesions, flat atypias and ductal hyperplasias of usual type were weakly positive for VEGF and associated to a low degree of neovascularization while in all in situ ductal carcinomas VEGF was highly expressed (Fig. 5A and 5B).

In the context of the above analysis, our results support the thesis that as the level of aggressiveness increases in the DCIS group from low to high, CD34 seems to be gradually down-regulated in the adjacent stroma while SMA seems to be gradually up-regulated. It has been suggested that this phenomenon might represent a change from a multipotent mesenchymal cell to a committed cell type [1]. Such an altered phenotype might be associated with a more favorable environment for the carcinoma, as far as possible infiltration and dissemination are concerned. In the present work, this alteration in the stroma phenotype was not evident in other hyperplastic and pre-malignant lesions such as LCIS or breast atypias which are thought to be less likely to progress to invasion [1].

## Conclusion

Our findings suggest that angiogenesis is observed before any significant fibroblastic stromagenesis in pre-invasive breast lesions. A composite phenotype characterized by VEGF positive epithelial cells and SMA positive/CD34 negative stromal cells, is identified mostly in intermediate and high grade in situ ductal carcinomas. This observation might imply for new therapeutic strategies to prevent the progression of DCIS to invasive carcinoma, using both anti-angiogenic factors and factors selectively targeting tumor stroma.

## List of abbreviations

Vascular Endothelial Growth Factor (VEGF), Smooth Muscle Actin (SMA), Microvessel Density (MVD), Ductal carcinoma in situ (DCIS), Metalloproteinases (MMPs), Fibroblast activating factor (FAP), Extracellular matrix (ECM), Connecting tissue growth factor (CTGF), Transforming growth factor beta-1 (TGF beta1), Low grade ductal carcinoma in situ (DCIS-L), Intermediate grade ductal carcinoma in situ (DCIS-I), High grade ductal carcinoma in situ (DCIS-H), Lobular carcinoma in situ (LCIS), Atypical lobular hyperplasia (ALH), Ductal epithelial hyperplasia of usual type (HUT), Flat atypia (FA), Loss of heterozygocity (LOH)

## Competing interests

The author(s) declare that they have no competing interests.

## Authors' contributions

KP conceived the idea, designed the study, evaluated the immunohistochemical staining, diagnosed the cases and wrote the main body of manuscript. IM diagnosed the cases, evaluated the immunohistochemical staining and carried out the angiogenesis evaluation. TV carried out the analysis of the results and contributed to manuscript drafting. PY diagnosed the cases and evaluated the immunohistochemical staining. DK, NL and TL were the attending physicians, collected the samples and revised the manuscript. ENS was supervising and coordinating the team, interpreted the results and critically revised the manuscript. All authors have read and approved the final manuscript.

## Pre-publication history

The pre-publication history for this paper can be accessed here:


